# Supplementary material for: Efficient industrial-current-density acetylene to polymer-grade ethylene via hydrogen-localization transfer over fluorine-modified copper
Source: Nat Commun. 2023 Dec 16;14:8384. doi: 10.1038/s41467-023-44171-5 (PMC10725425; doi:10.1038/s41467-023-44171-5)
Supplement: Supplementary file 1 — Supplementary Information [file 41467_2023_44171_MOESM1_ESM.pdf]

Supporting information

Efficient Industrial-Current-Density Acetylene to Polymer-Grade Ethylene via Hydrogen-Localization Transfer over Fluorine-Modified Copper

Lei Bai[1], Yi Wang[1], Zheng Han[1], Jinbo Bai[2], Kunyue Leng[1]*, Lirong Zheng[3*], Yunteng Qu[1]* and Yuen Wu [4]

[1]*International Collaborative Center on Photoelectric Technology and Nano Functional Materials, Institute of Photonics and Photon-Technology, Northwest University, Xi'an, Shaanxi 710069, China*

[2]*Université Paris-Saclay, CentraleSupélec, ENS Paris-Saclay, CNRS, LMPS-Laboratoire de Mécanique Paris-Saclay, 8-10 rue Joliot-Curie, Gif-sur-Yvette 91190, France*

[3]*Institute of High Energy Physics, Beijing 100039, China*

[4]*School of Chemistry and Materials Science, University of science and Technology of China, Hefei 230026, China*

Keywords: Acetylene semihydrogenation, Electrocatalyst, Ethylene production

Corresponding Authors: Kunyue Leng (lengky@nwu.edu.cn), Lirong Zheng (zhenglr@ihep.ac.cn), Yunteng Qu (yuntengqu@nwu.edu.cn)

Supplementary Figures and Tables

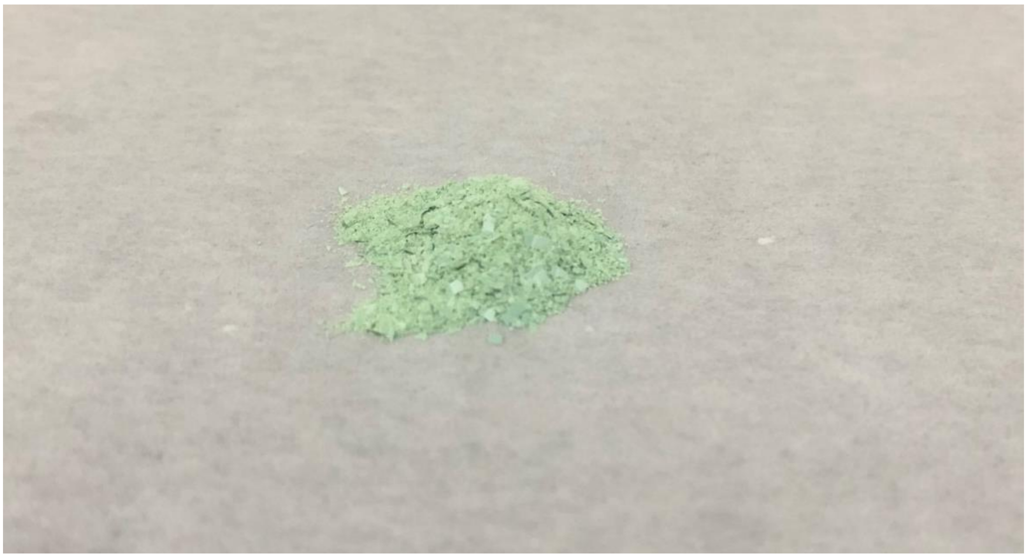

Figure S1. Digital paragraph of Cu(OH)F, which takes an appearance of light-green slice and powder.

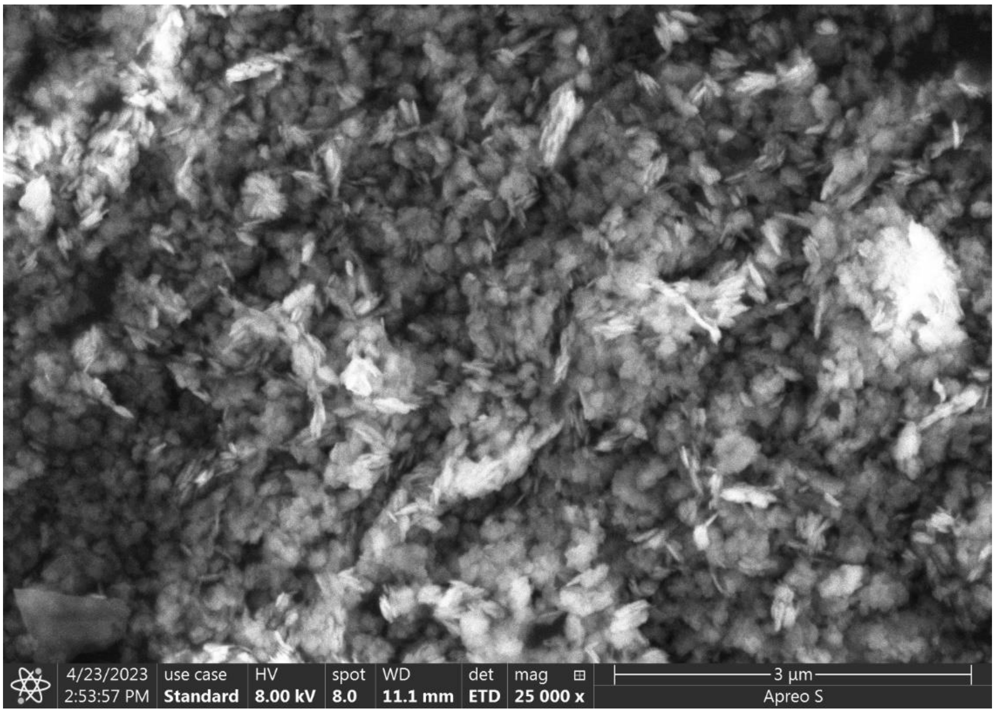

Figure S2. SEM image of Cu(OH)F. The as-prepared Cu(OH)F exhibits a morphology of the accumulation of nanosheets.

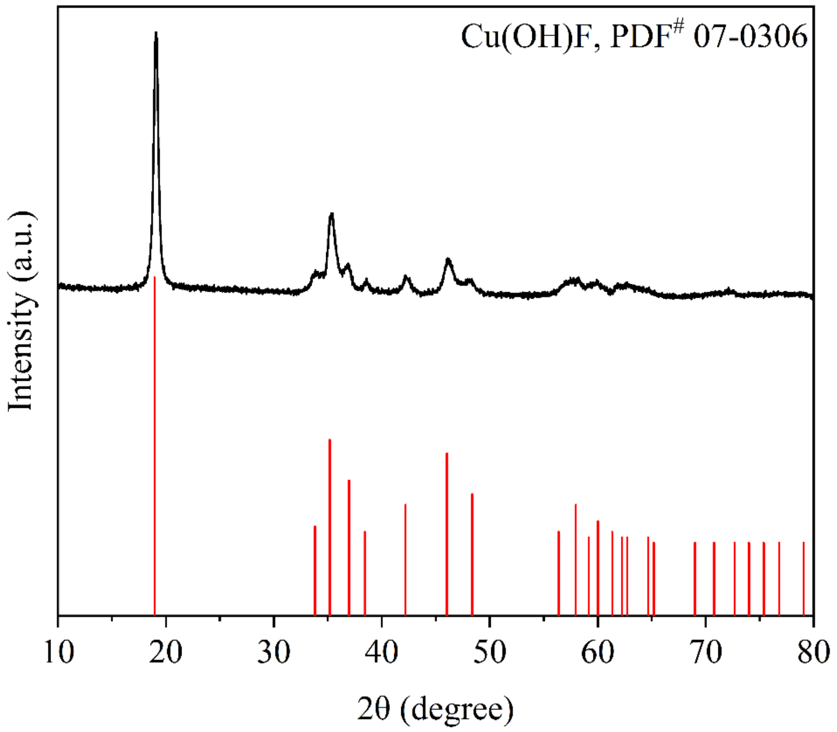

Figure S3. XRD pattern of Cu(OH)F. The characteristic diffraction peaks at 18.9°, 35.2°, 36.9°, 38.4°, 46.0° are consistent with the patterns of the crystalline Cu(OH)F.

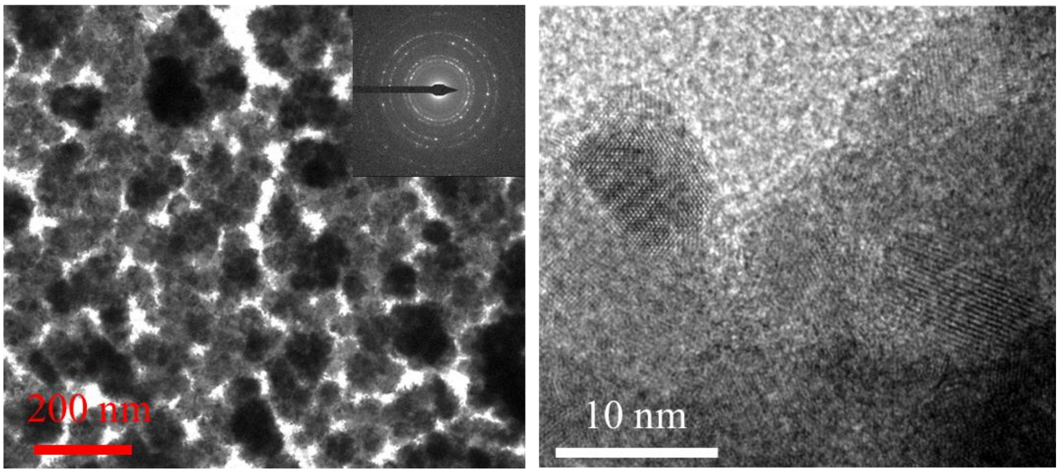

Figure S4. TEM images of Cu-F. The Cu-F reveals the aggregation of irregular nanoparticles with a size ranging from 45-90 nm, and the lattice spacing are measured at 0.208 nm that corresponding to the Cu (111) plane.

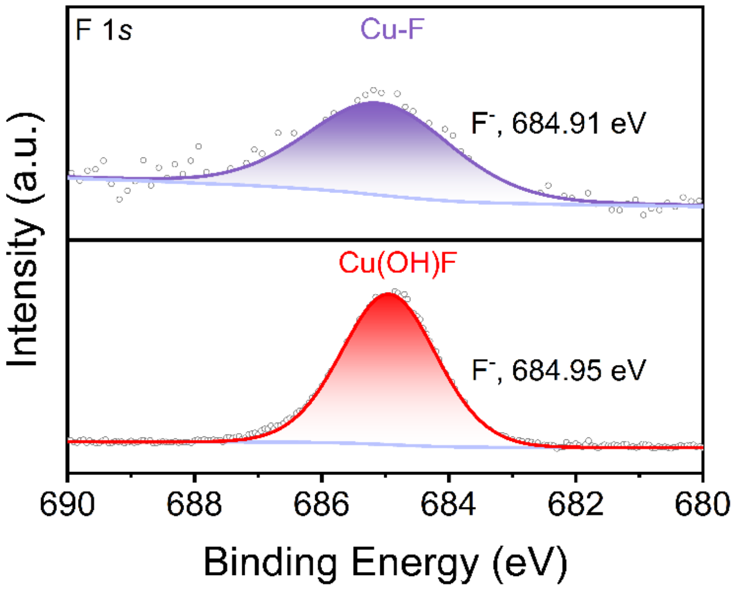

Figure S5. F 1*s* XPS spectra of Cu-F and Cu(OH)F. The Cu (OH)F and Cu-F shows similar binding energy for F 1*s*, indicating the adsorbed F in Cu-F.

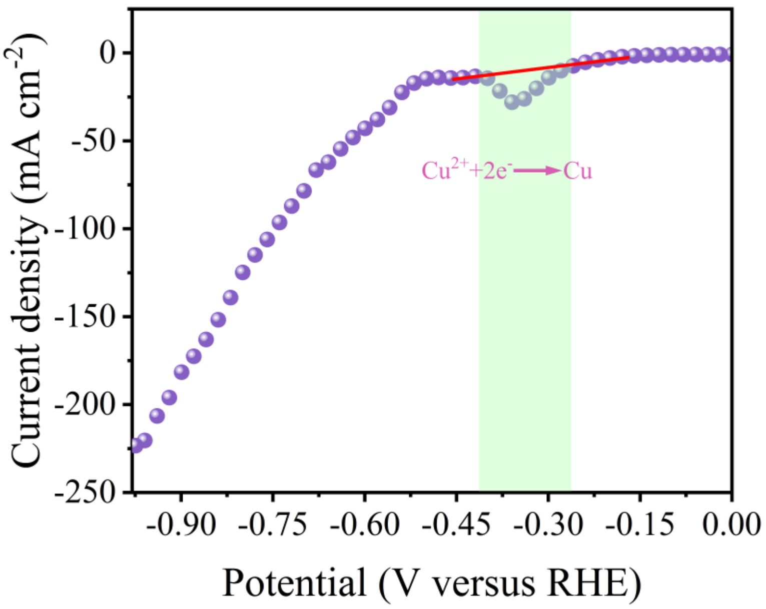

Figure S6. The LSV curve of the Cu(OH)F reduction recorded in 1 M KOH with a Ar flow (30 ml min^{-1}) and a scan rate of 10 mV s^{-1}.

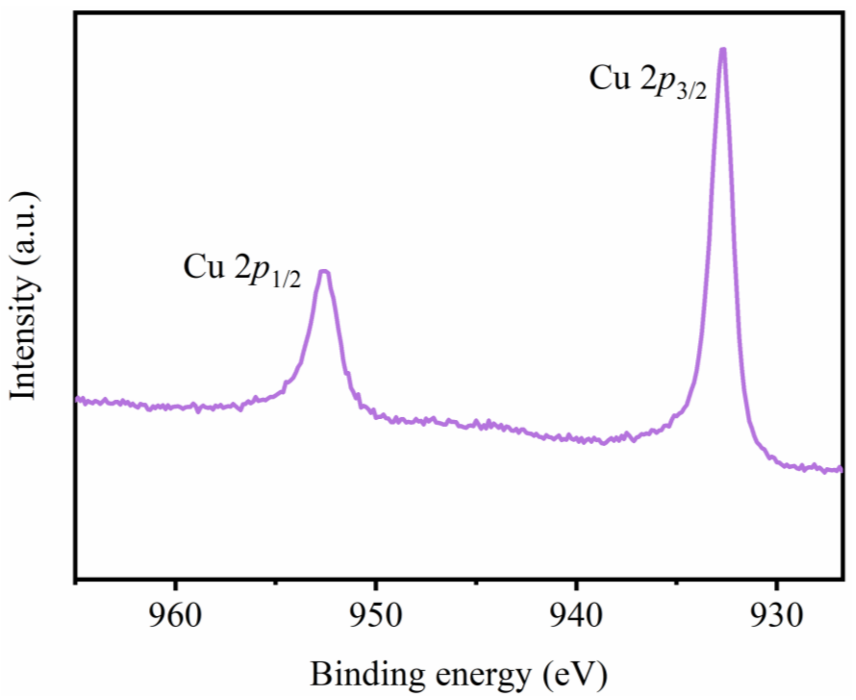

Figure S7. Cu 2*p* XPS spectra of Cu-F. Cu-F exhibits a binding energy for Cu $2p_{3/2}$ at 932.7 eV, slightly higher than the 932.4 eV for Cu^0.

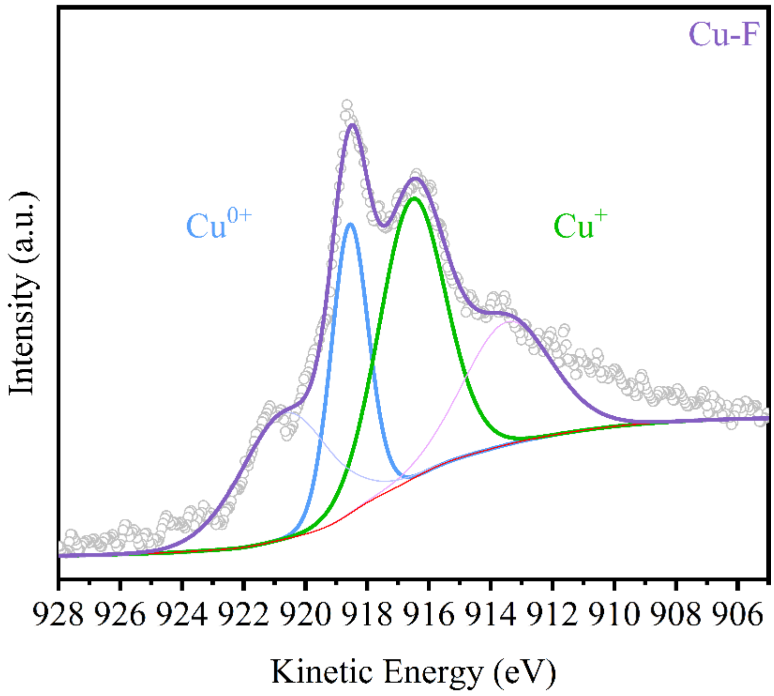

Figure S8. LMM spectra of Cu-F, which indicates the co-existence of Cu^{0+} and Cu^{+} in Cu-F

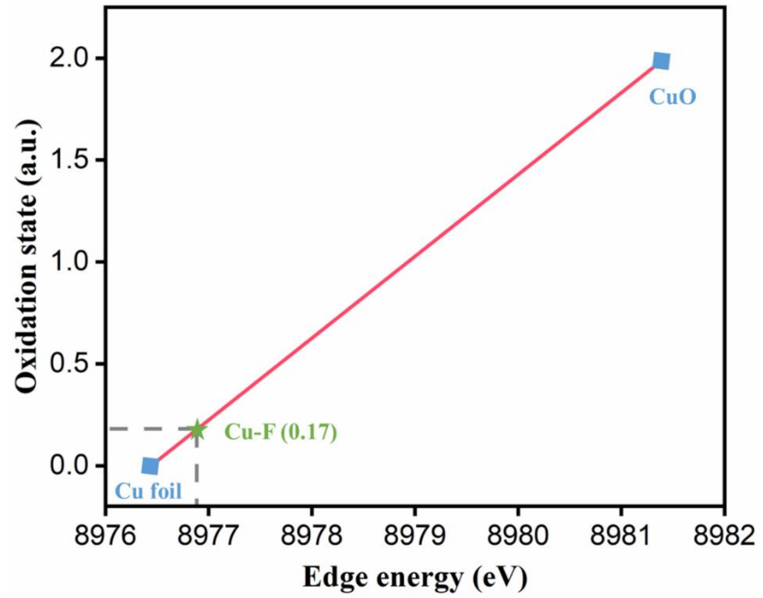

Figure S9. The simulative oxidation state of Cu in Cu-F based on the edge energy. The average oxidation state of Cu in Cu-F is measured approximately to +0.2.

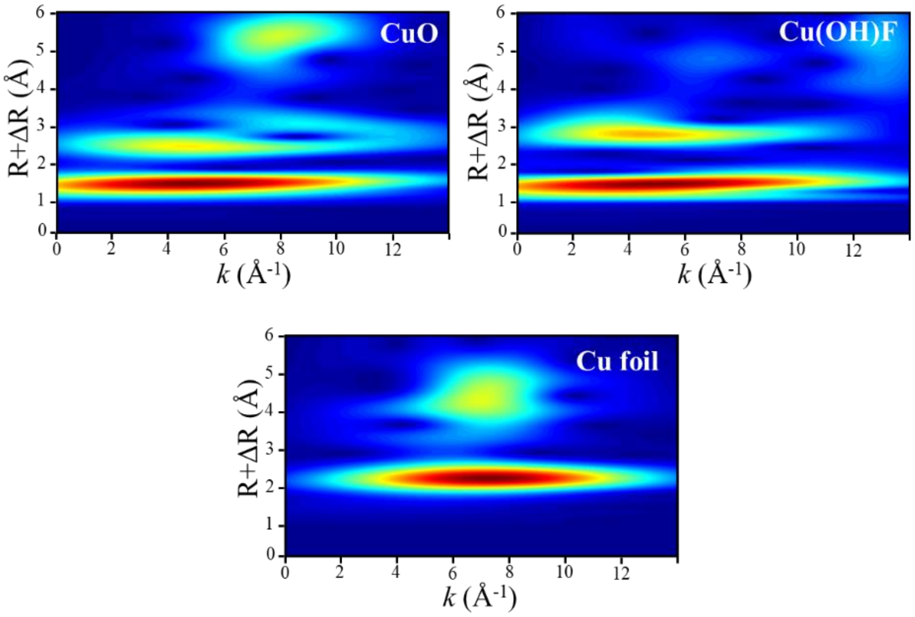

Figure S10. Wavelet transformations of CuO, Cu(OH)F and Cu foil. This observation in agreement with the FT EXAFS spectra in Figure 1g, indicating the partial coordination of Cu with F in the Cu-F catalyst.

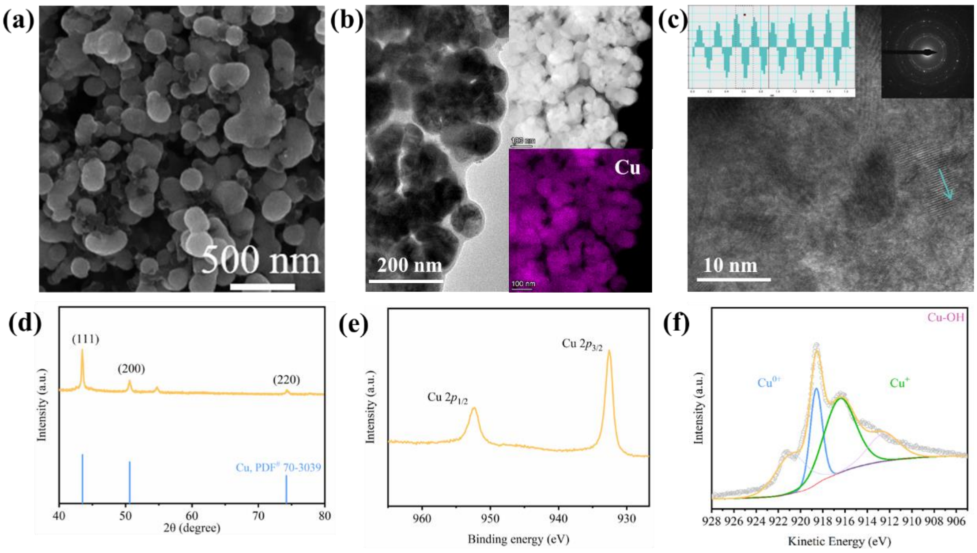

Figure S11. Characterization of Cu NP. (a) SEM image. (b) TEM image and element mapping. (c) HR-TEM image. (d) XRD pattern. (e) Cu 2*p* XPS spectrum. (f) LMM spectrum.

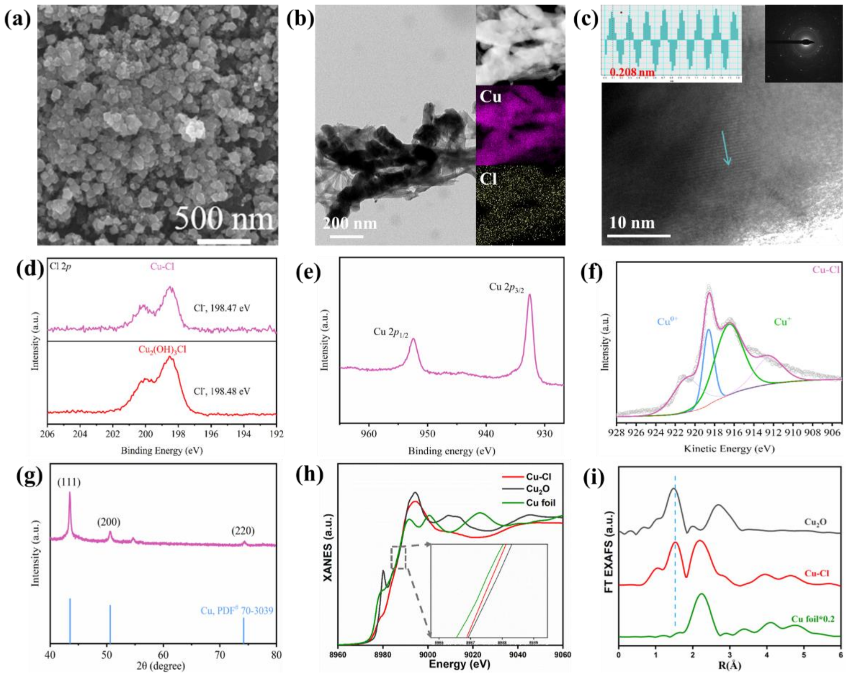

Figure S12. Characterization of Cu-Cl. (a) SEM image. (b) TEM image and element mapping. (c) HR-TEM image. (d) Cl 2p XPS spectra. (e) Cu 2*p* XPS spectrum. (f) LMM spectrum. (g) XRD pattern. (h) Cu k-edge XANES spectra. (i) Cu k-edge FT EXAFS spectra.

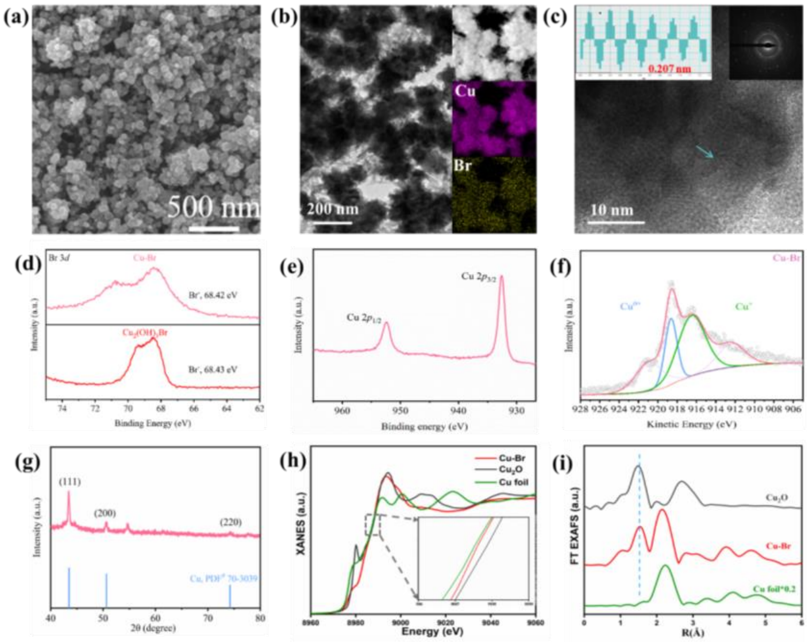

Figure S13. Characterization of Cu-Br. (a) SEM image. (b) TEM image and element mapping. (c) HR-TEM image. (d) Br 3d XPS spectra. (e) Cu 2*p* XPS spectrum. (f) LMM spectrum. (g) XRD pattern. (h) Cu k-edge XANES spectra. (i) Cu k-edge FT EXAFS spectra.

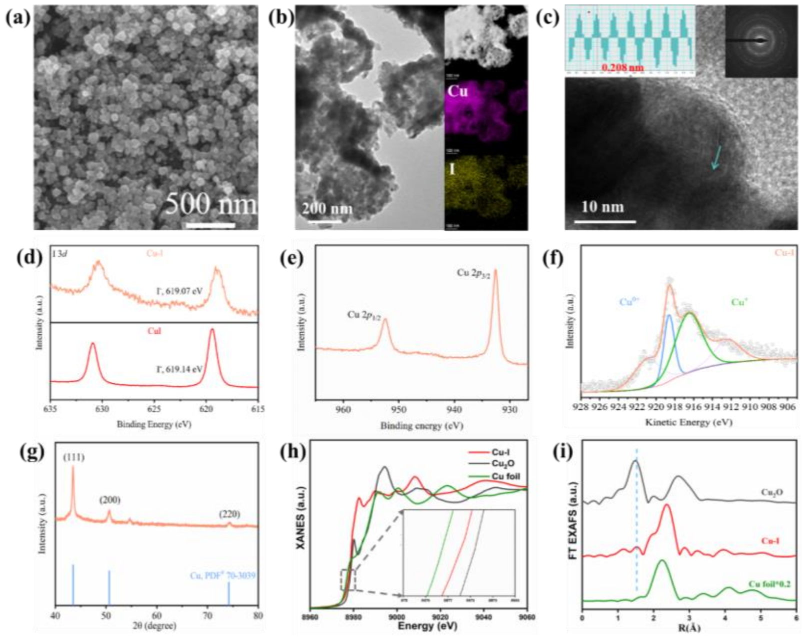

Figure S14. Characterization of Cu-I. (a) SEM image. (b) TEM image and element mapping. (c) HR-TEM image. (d) I 3d XPS. (e) Cu 2*p* XPS spectra. (f) LMM spectrum. (g) XRD pattern. (h) Cu k-edge XANES spectra. (i) Cu k-edge FT EXAFS spectra.

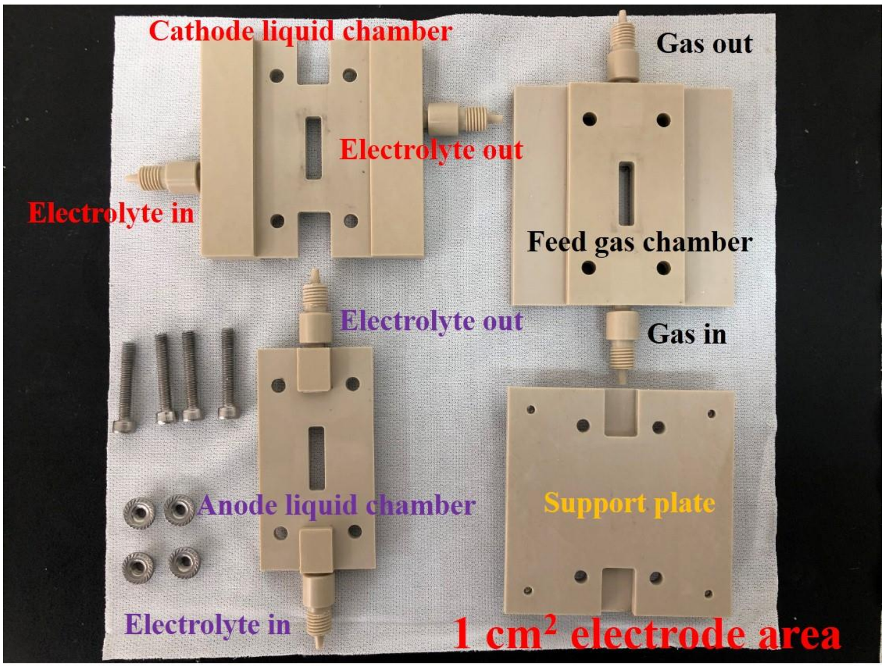

Figure S15. Digital graph of the 1 cm^2 flow cell, which is mainly consisted by cathode liquid chamber, anode liquid chamber, feed gas chamber and support plate.

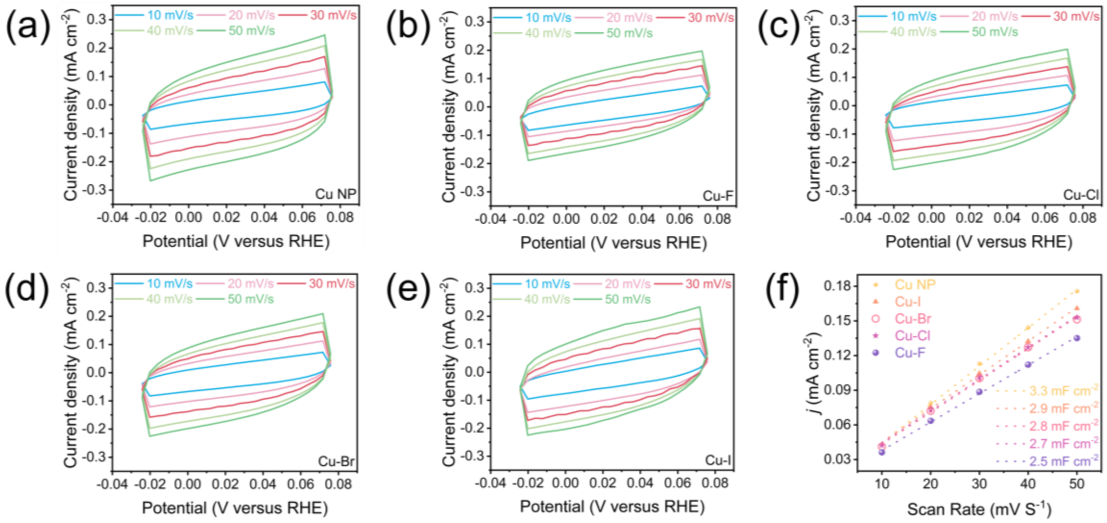

Figure S16. Electrochemical capacitance measurements. Cyclic voltammogram curves of (a) Cu NP, (b) Cu-F, (c) Cu-Cl, (d) Cu-Br and (e) Cu-I at different scan rate. (f) The corresponding charging current densities vs. applied scan rate.

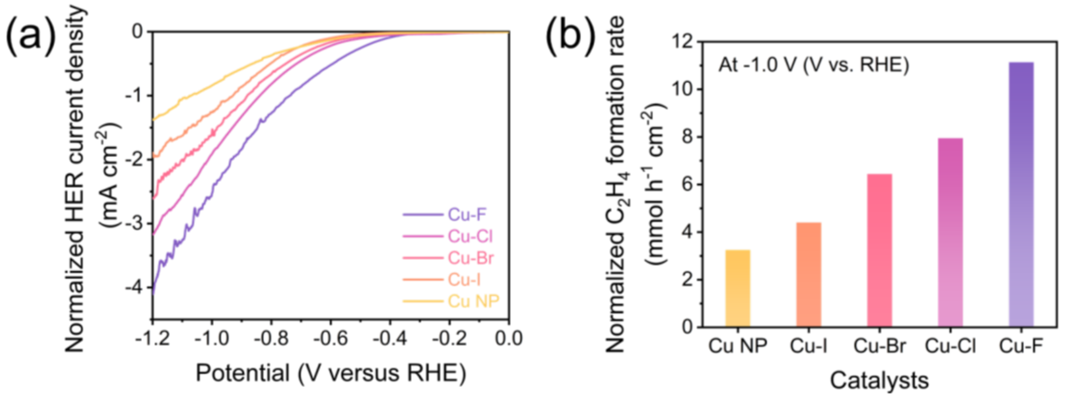

Figure S17. ECSA-normalized electrocatalytic performance of various catalysts. (a) LSV curves of HER measured under Ar flow. (b) C_2H_4 formation rate at -1.0 V measured under 70 mol% C_2H_2/Ar flow. Measured using a three-electrode flow cell (1 cm^2) in 1 M KOH at room temperature with gas flow rate of 30 ml min^{-1}. The results are presented without iR compensation.

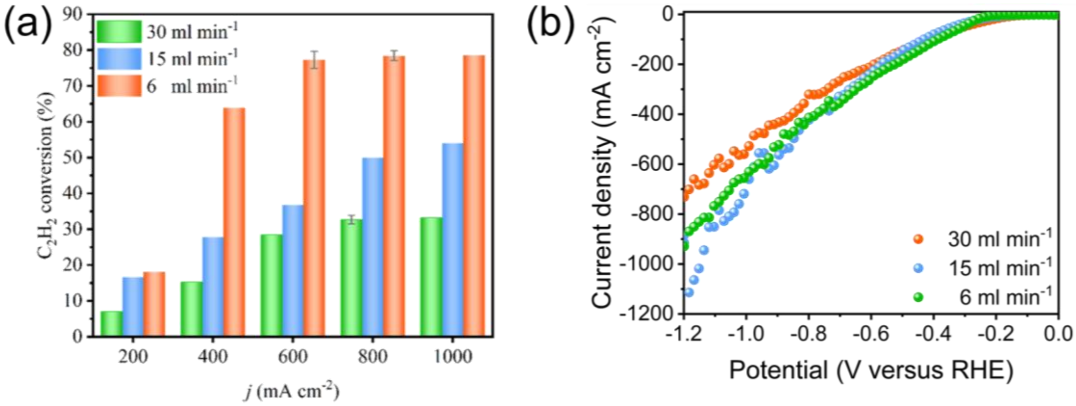

Figure S18. ESAE performance of Cu-F at different flow rate of the feed gas. (a) Single-path C_2H_2 conversion vs. current density, the maximum measurement error is ± 4.6%. (b) LSV curves. Measured using a three-electrode flow cell (1 cm^2) in 1 M KOH at room temperature under 70 mol% C_2H_2/Ar flow. The results are presented without iR compensation.

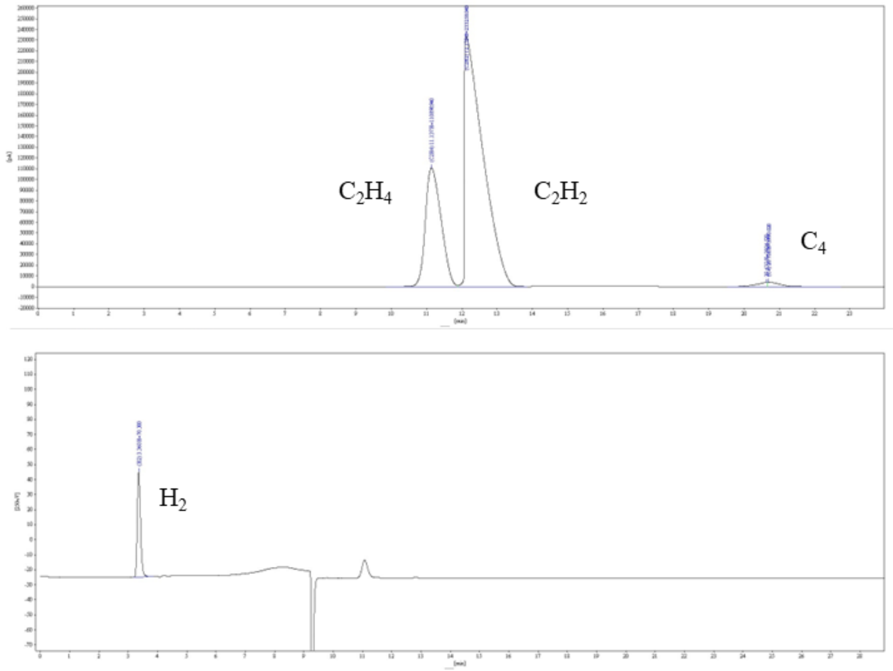

Figure S19. Gas chromatography analysis of the outlet gas from flow-cell. Ethylene (C_2H_4) is identified as the main production, accompanied by few H_2 and C_4.

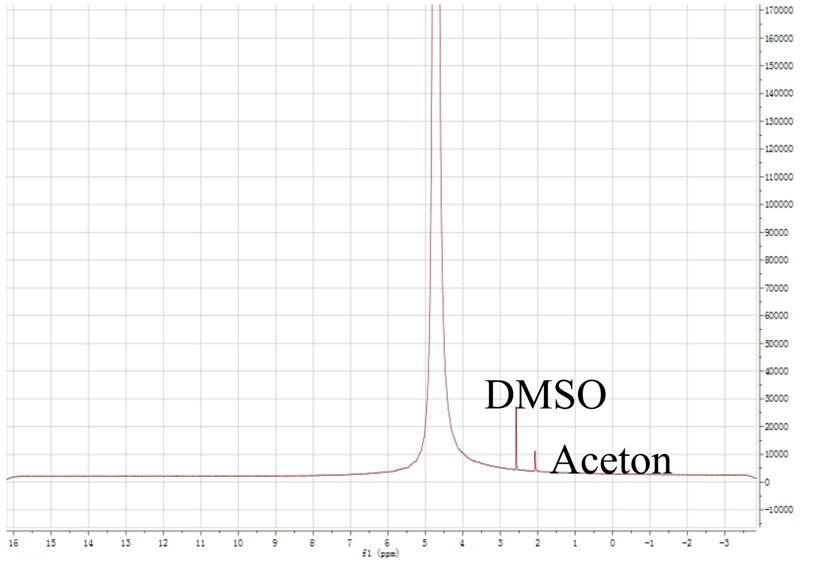

Figure S20. Liquid NMR analysis of the electrolyte after the ESAE evaluation in flow-cell. Acetone origins from C_2H_2 feed gas. No liquid production is detected in the used electrolyte.

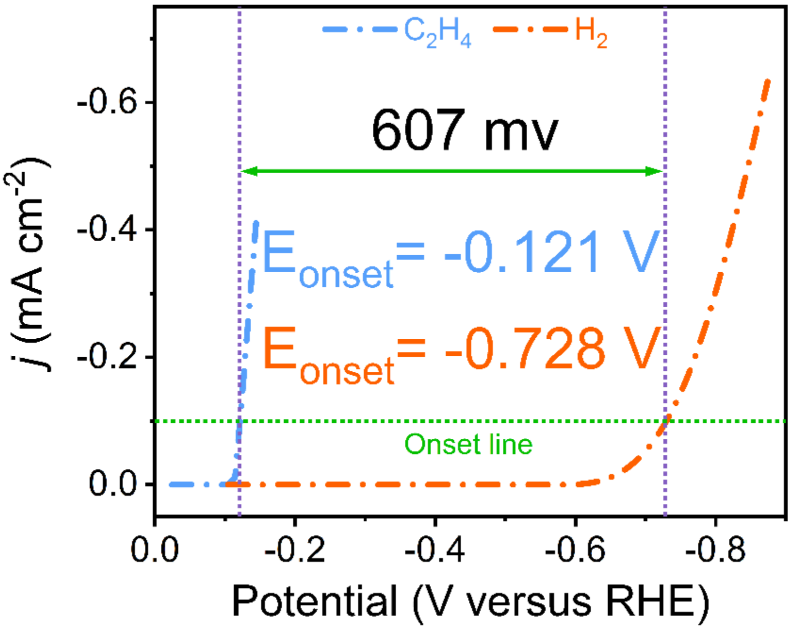

Figure S21. The onset potentials over Cu-F for C_2H_4 and H_2 generation. Measured using a three-electrode flow cell (1 cm^2) in 1 M KOH at room temperature under 70 mol% C_2H_2/Ar flow (30 ml min^{-1}). The results are presented without iR compensation.

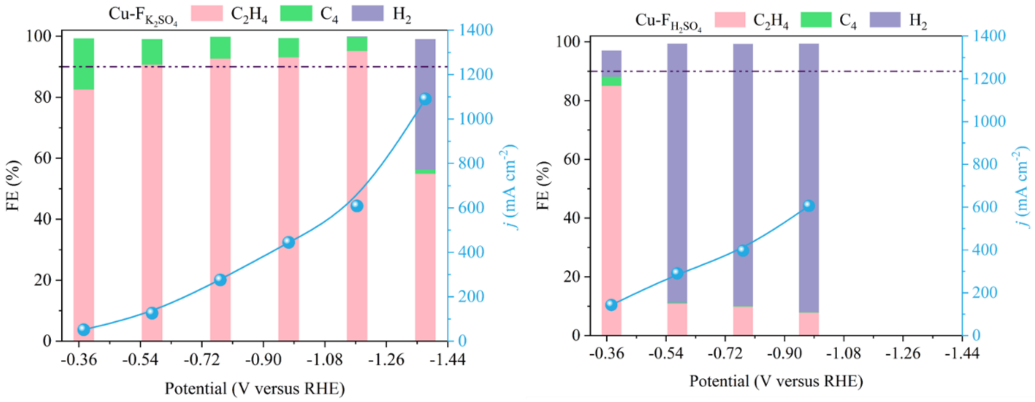

Figure S22. Faradaic efficiency of the ESAE productions vs. applied potential over Cu-F in neutral and acidic medium. Measured using a three-electrode flow cell (1 cm^2) in 0.5 M K_2SO_4 or H_2SO_4 at room temperature under 70 mol% C_2H_2/Ar flow (30 ml min^{-1}). The results are presented without iR compensation.

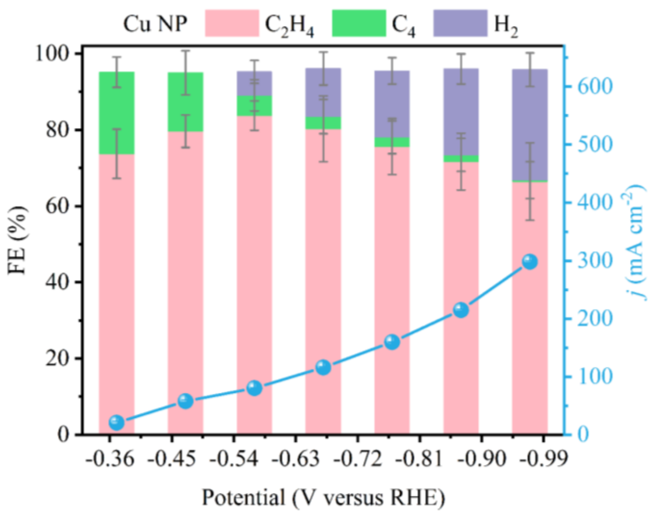

Figure S23. Faradaic efficiency of the ESAE productions vs. applied potential over Cu NP. Measured using a three-electrode flow cell (1 cm^2) in 1 M KOH at room temperature under 70 mol% C_2H_2/Ar flow (30 ml min^{-1}). The results are presented without iR compensation. the maximum measurement error is ±4.8%.

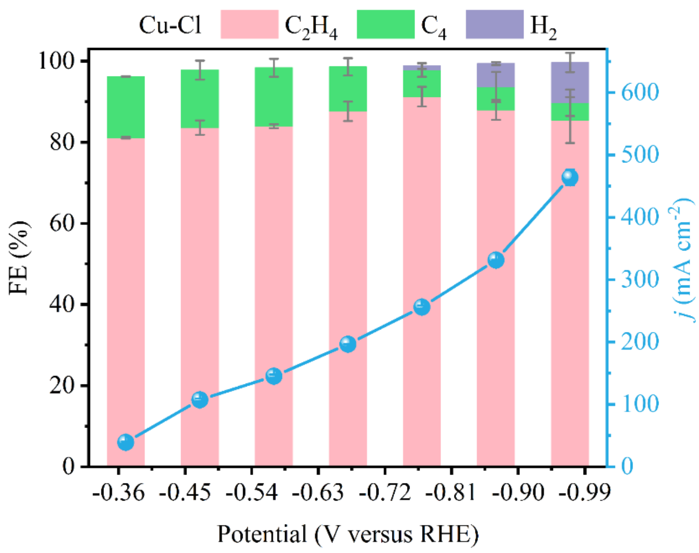

Figure S24. Faradaic efficiency of the ESAE productions vs. applied potential over Cu-Cl. Measured using a three-electrode flow cell (1 cm^2) in 1 M KOH at room temperature under 70 mol% C_2H_2/Ar flow (30 ml min^{-1}). The results are presented without iR compensation. The maximum measurement error is ±3.4%.

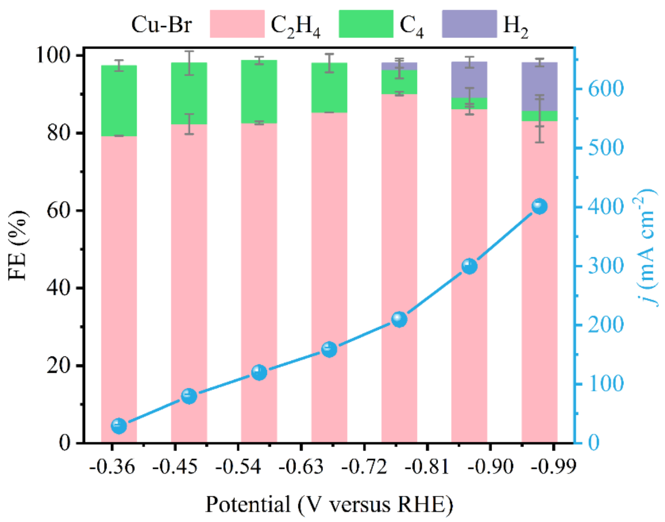

Figure S25. Faradaic efficiency of the ESAE productions vs. applied potential over Cu-Br. Measured using a three-electrode flow cell (1 cm^2) in 1 M KOH at room temperature under 70 mol% C_2H_2/Ar flow (30 ml min^{-1}). The results are presented without iR compensation. The maximum measurement error is ±4.1%.

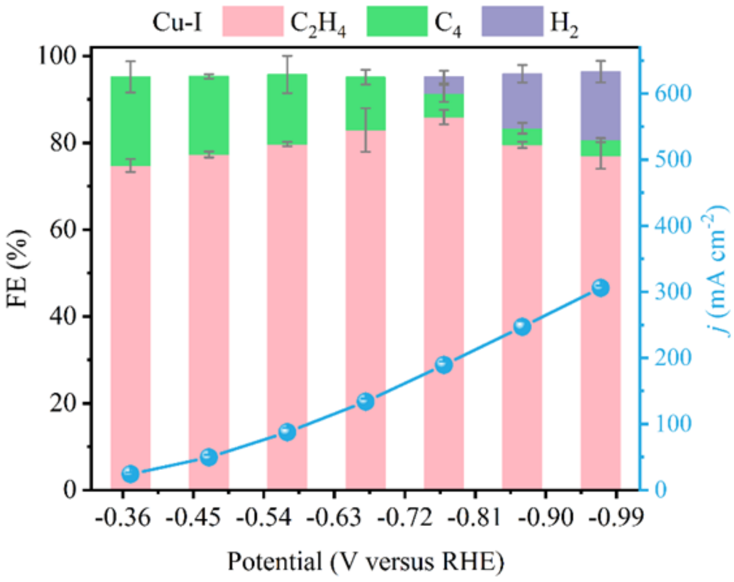

Figure S26. Faradaic efficiency of the ESAE productions vs. applied potential over Cu-I. Measured using a three-electrode flow cell (1 cm^2) in 1 M KOH at room temperature under 70 mol% C_2H_2/Ar flow (30 ml min^{-1}). The results are presented without iR compensation. The maximum measurement error is ±4.2%.

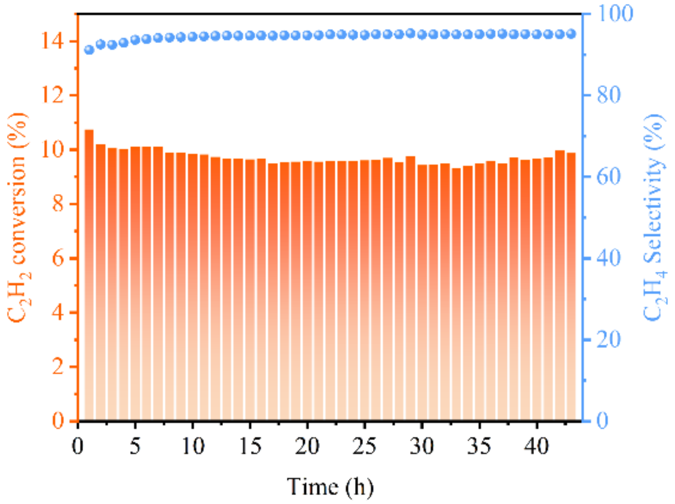

Figure S27. C_2H_2 conversion and C_2H_4 selectivity of Cu-F vs. reaction time in the long-term stability test. Faradaic efficiency of the ESAE productions vs. applied potential over Cu NP. Measured using a three-electrode flow cell (1 cm^2) in 1 M KOH at room temperature under 70 mol% C_2H_2/Ar flow (30 ml min^{-1}). A constant current density is set at 200 mA cm^{-2}.

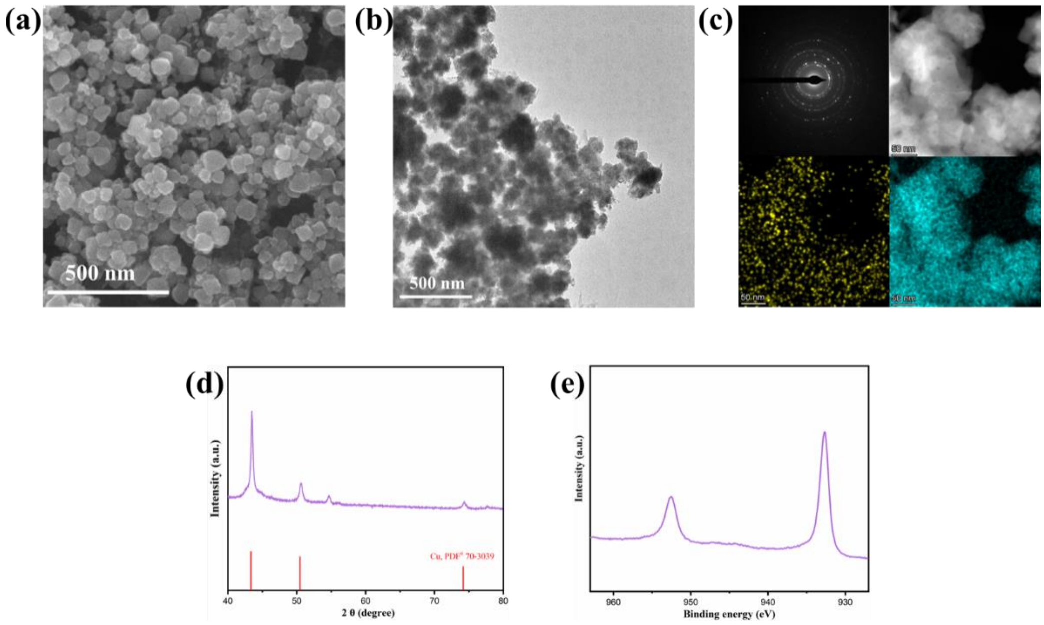

Figure S28. Characterizations of the Cu-F after long-term stability test. (a) SEM image. (b) TEM image. (c) SAED and element mapping. (d) XRS pattern. (e) Cu 2*p* XPS spectrum.

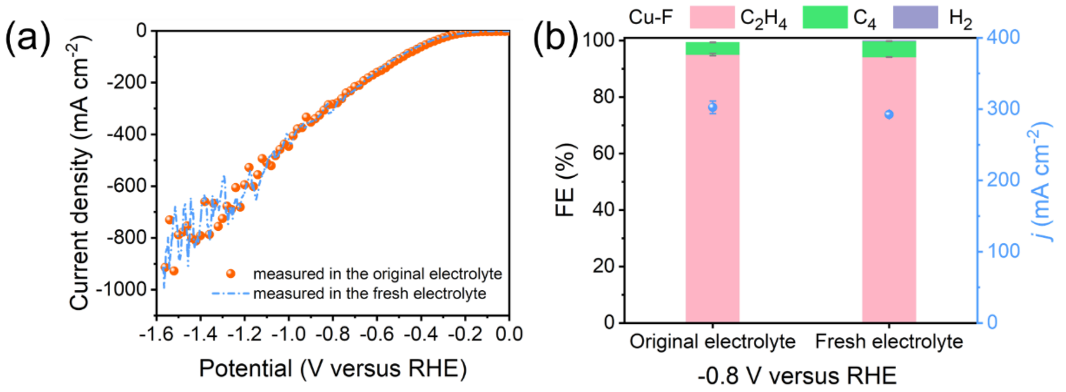

Figure S29. The effect of the leached F in the electrolyte. (a) LSV curves. The orange sphere represents the curve measured in the original electrolyte used for generating Cu-F, which may contain the leached F^-; The bule line represents the electrolyte is replaced by a fresh one after the generation of Cu-F. (b) FE and the current density, the maximum measurement error is ±2.8%.

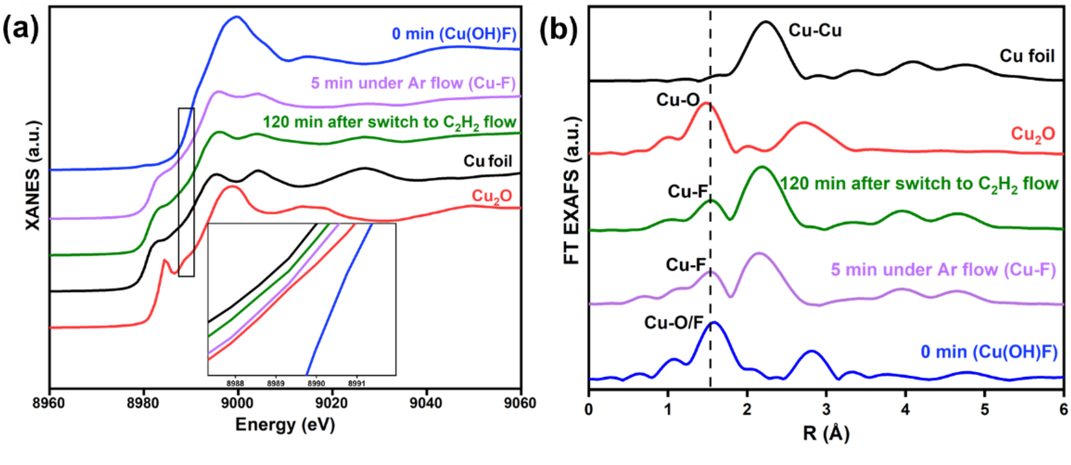

Figure S30. The in-situ XAFS measured on the generation process of Cu-F and the subsequent acetylene semi-hydrogenation process. (a) The Cu k-edge XANES spectra. (b) Cu k-edge FT-EXAFS spectra. Measured at -0.6 V vs. RHE (-1.6 V vs. Ag/AgCl) in different feed gas at different time.

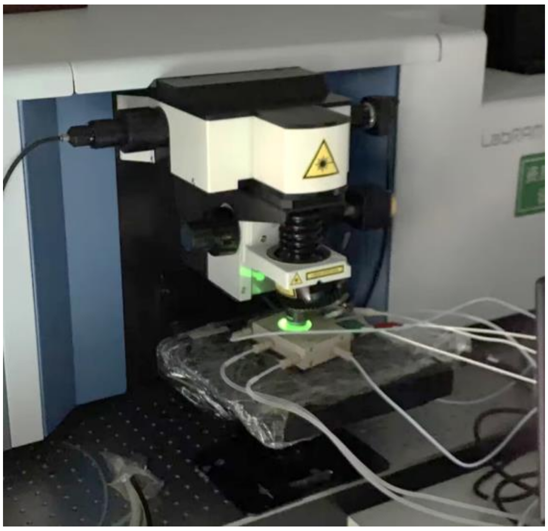

Figure S31. The operando Raman device used in this work. A three-electrode observable window electrochemical cell with a counter electrode of carbon rod and Ag/AgCl under controlled potentials in 1 M KOH electrolyte, and a controlled active area by an insulation layer on carbon paper sprayed with 1 mg Cu(OH)F was used as the working electrode.

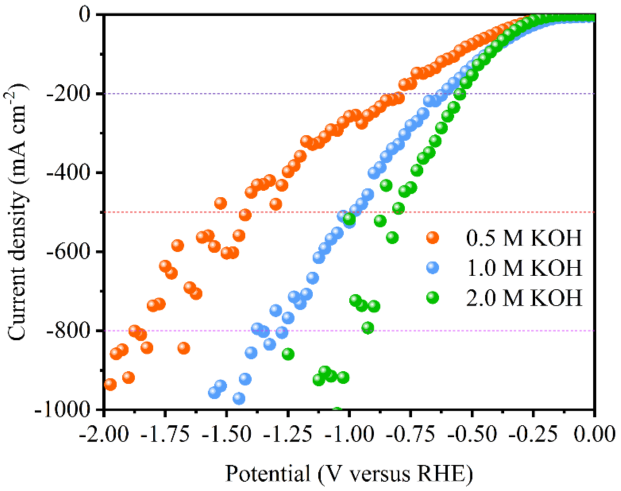

Figure S32. The LSV curves of Cu-F in KOH solution with different concentration. Measured using a three-electrode flow cell (1 cm^2) at room temperature under 70 mol% C_2H_2/Ar flow (30 ml min^{-1}). The results are presented without iR compensation.

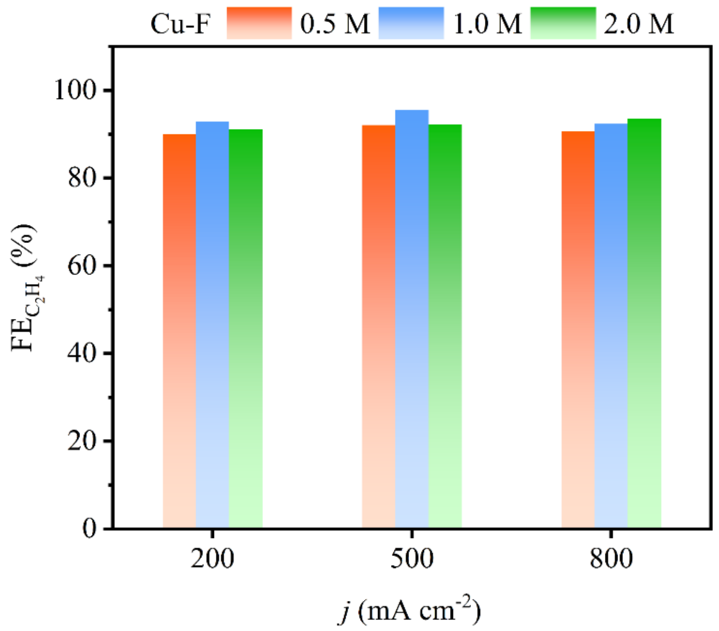

Figure S33. The effect of the KOH concentration on the FE over Cu-F. Measured using a three-electrode flow cell (1 cm^2) at room temperature under 70 mol% C_2H_2/Ar flow (30 ml min^{-1}). The results are presented without iR compensation.

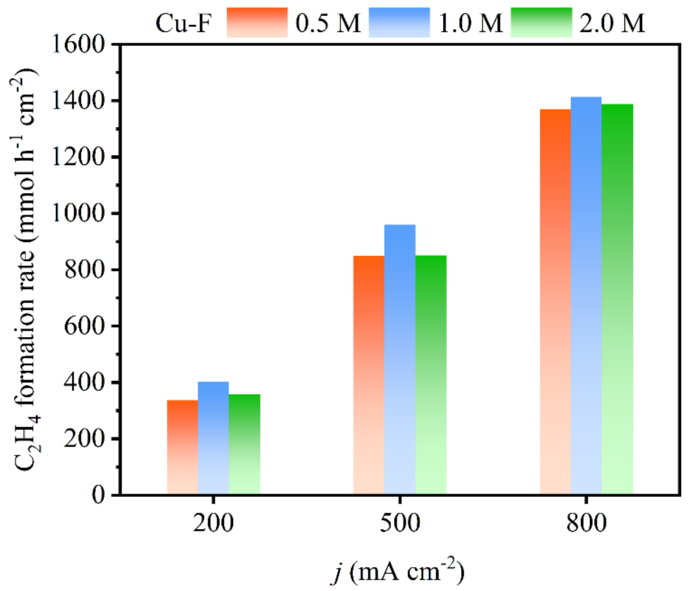

Figure S34. The effect of the KOH concentration on the C_2H_4 formation rate over Cu-F. Measured using a three-electrode flow cell (1 cm^2) at room temperature under 70 mol% C_2H_2/Ar flow (30 ml min^{-1}). The results are presented without iR compensation.

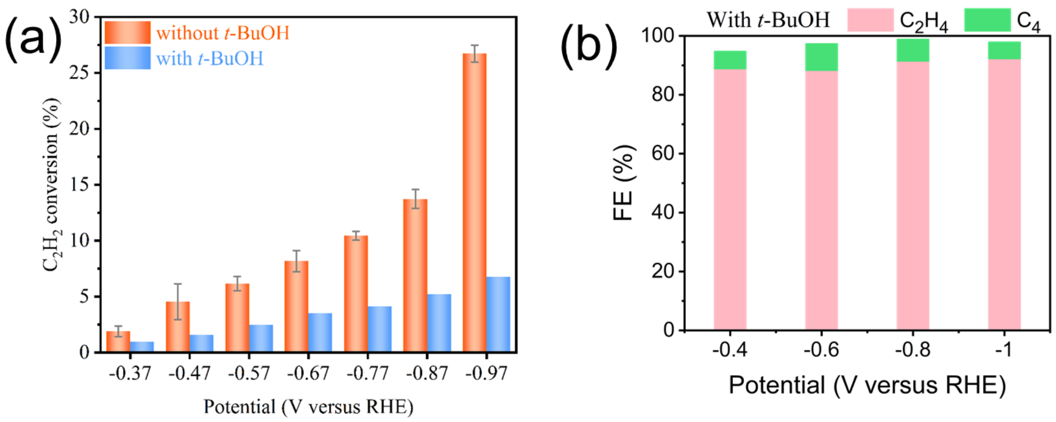

Figure S35. (a) Potential-dependent C_2H_2 conversion change over Cu-F with or without the addition of tert-Butanol in the electrolyte, the maximum measurement error is ±4.8%. (b) FE of Cu-F with tert-Butanol in the electrolyte. Measured using a three-electrode flow cell (1 cm^2) in 1 M KOH at room temperature under 70 mol% C_2H_2/Ar flow (30 ml min^{-1}). The results are presented without iR compensation.

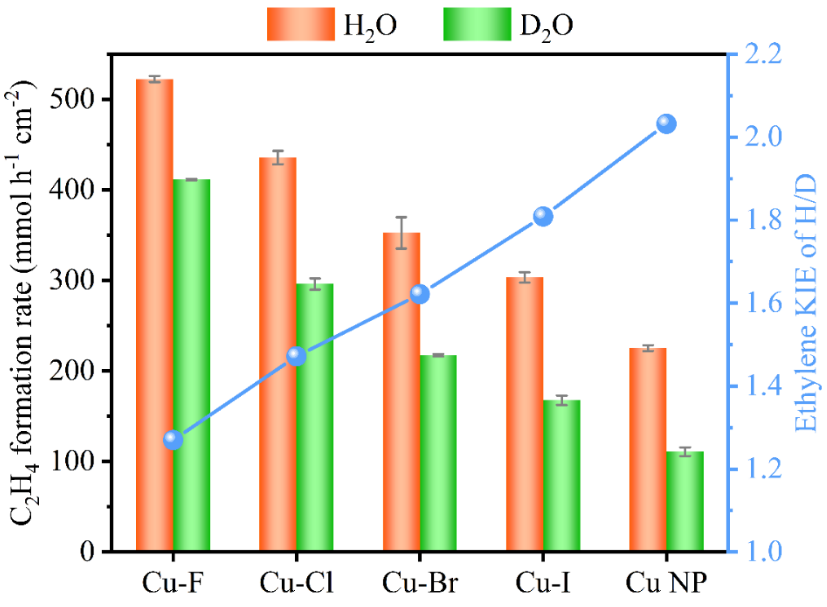

Figure S36. The KIE of Cu-F, Cu-Cl, Cu-Br, Cu-I and Cu NP. Measured using a three-electrode flow cell (1 cm^2) in 1 M KOH (H_2O and D_2O as solvent respectively) at room temperature under 70 mol% C_2H_2/Ar flow (30 ml min^{-1}). The results are presented without iR compensation. The maximum measurement error is ±4.9%.

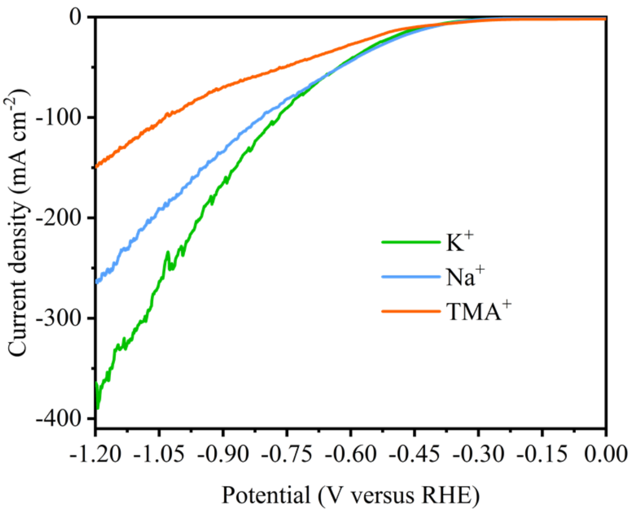

Figure S37. LSV curves in pure Ar over Cu-F with 1 M KOH, NaOH and TMAH electrolyte. Measured using a three-electrode flow cell (1 cm^2) at room temperature under Ar flow (30 ml min^{-1}). The results are presented without iR compensation.

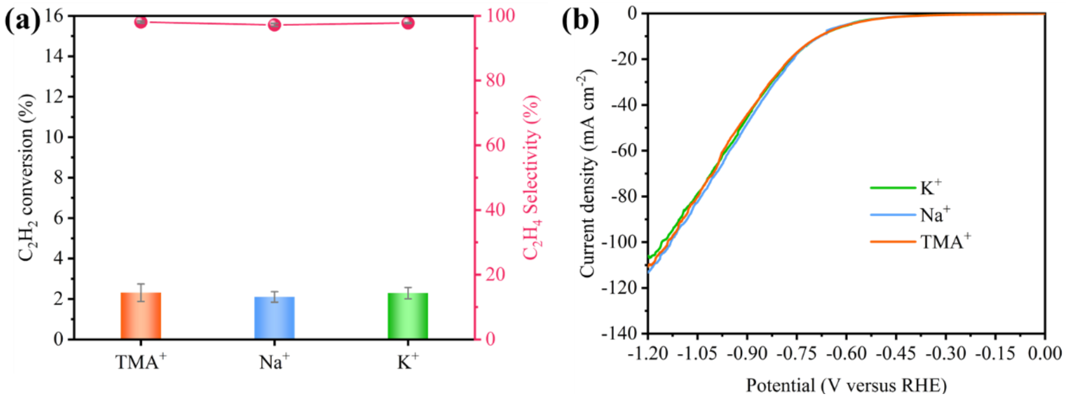

Figure S38. (a) C_2H_2 conversion measured under 70 % C_2H_2/Ar (30 ml min^{-1}) flow and (b) LSV measured under pure Ar flow (30 ml min^{-1}) over Cu NP in 1 M KOH, NaOH and TMAH electrolyte. Measured using a three-electrode flow cell (1 cm^2) at room temperature The results are presented without iR compensation. The maximum measurement error is ±3.6%.

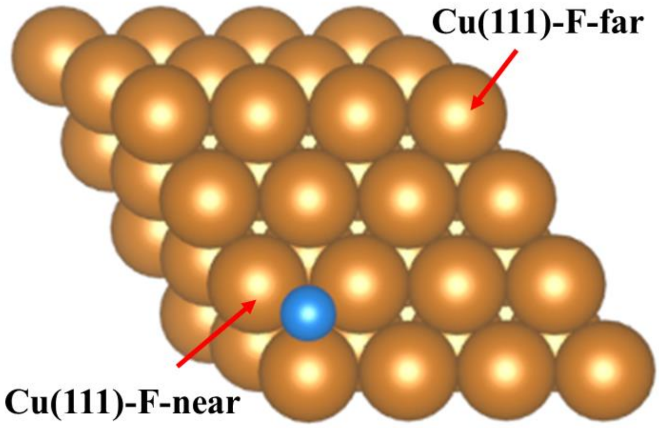

Figure S39. The simulative structure of the Cu sites near and far from the F atom in Cu-F.

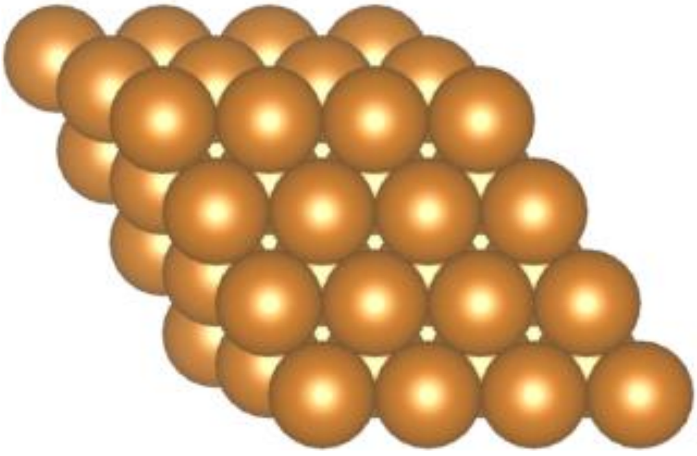

Figure S40. The simulative structure of the Cu (111), which displays the (111) plane of crystalline Cu, matching the physical characterizations of Cu-F.

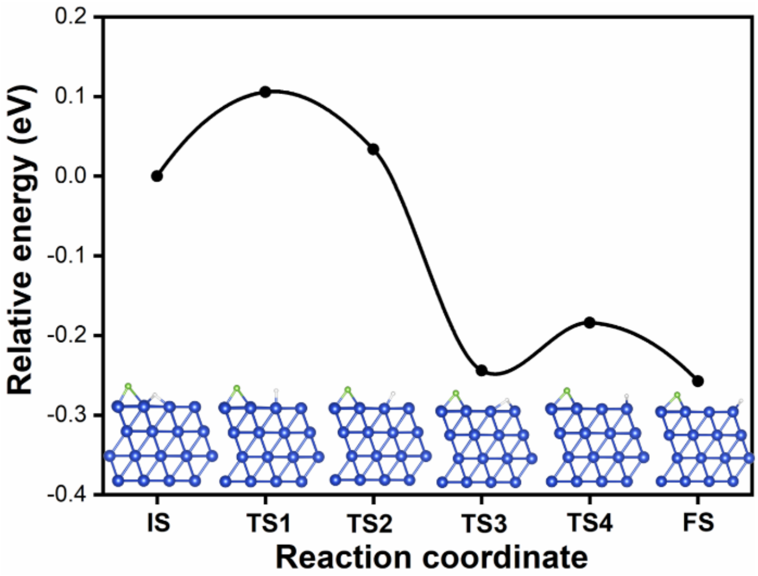

Figure S41. Energy of the system as function of the transfer pathway for a surface *H on Cu-F, calculating based on CINEB.

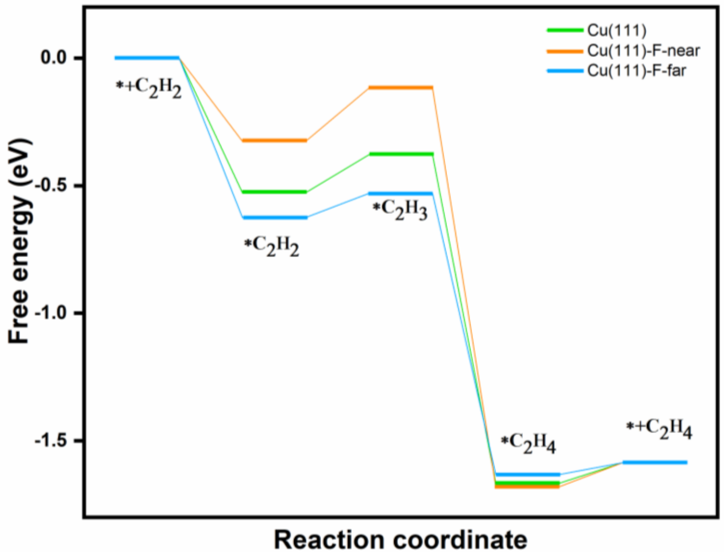

Figure S42. Free energy diagram for the hydrogenation of C_2H_2 at -1.0 V vs. RHE, which displays the similar trend with that at 0 V vs. RHE, but the reaction barrier is reduced.

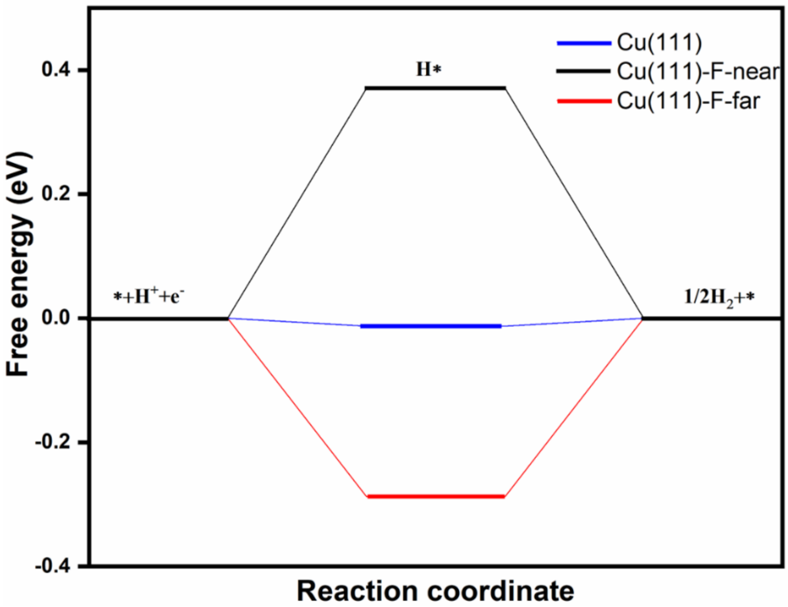

Figure S43. Free energy diagram for the hydrogen combination at 0 V vs. RHE. The H_2 generation barrier of Cu (111)-F-far is measured as 0.29 eV, higher than the C_2H_2 semi-hydronation barrier of 0.12 eV.

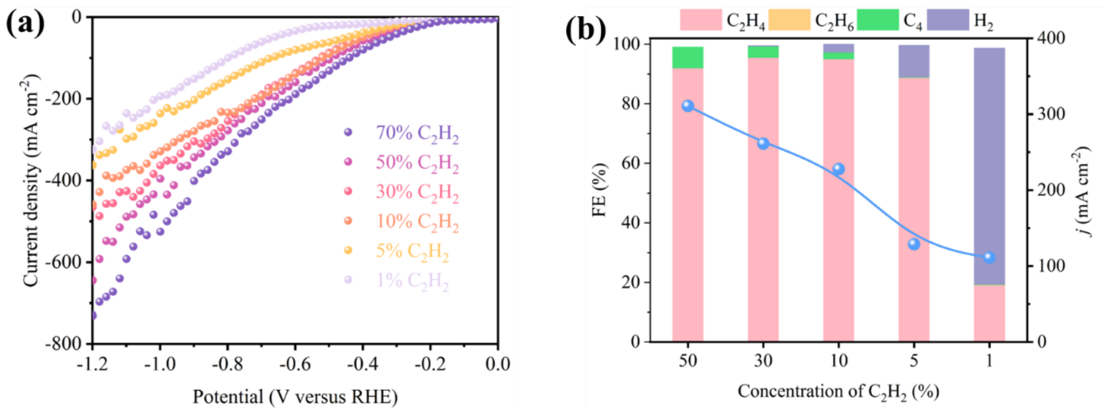

Figure S44. ESAE performance of Cu-F under C_2H_2/Ar flow with different C_2H_2 concentration (mol%, 30 ml min^{-1}). (a) LSV curves. (b) Faraday efficiency and current density at different C_2H_2 concentration. Measured using a three-electrode flow cell (1 cm^2) in 1 M KOH at room temperature. The results are presented without iR compensation.

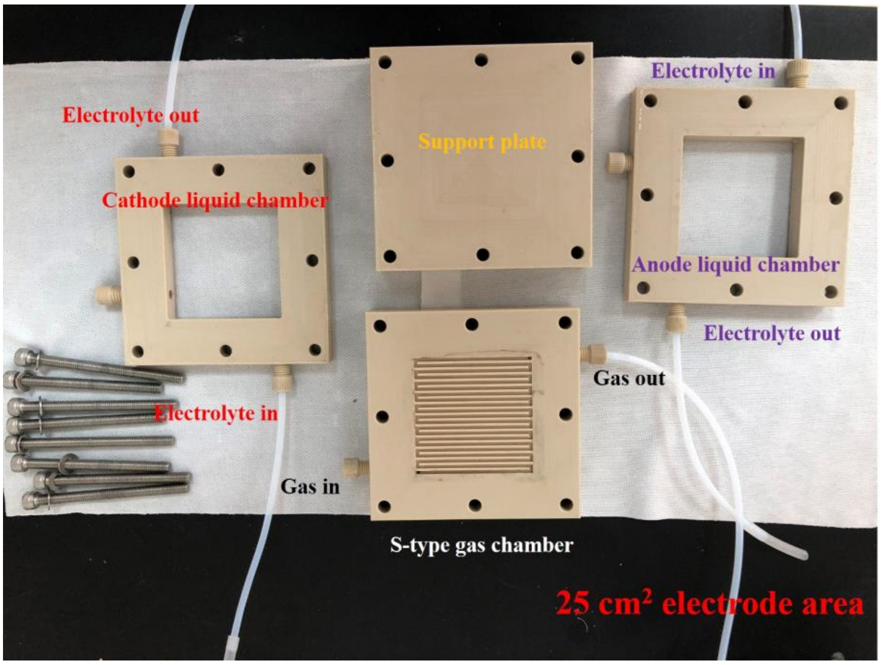

Figure S45. Digital image of the 25 cm^2 flow-cell with S type gas chamber, which is mainly consisted by cathode liquid chamber, support plate, anode liquid chamber and S-type gas chamber.

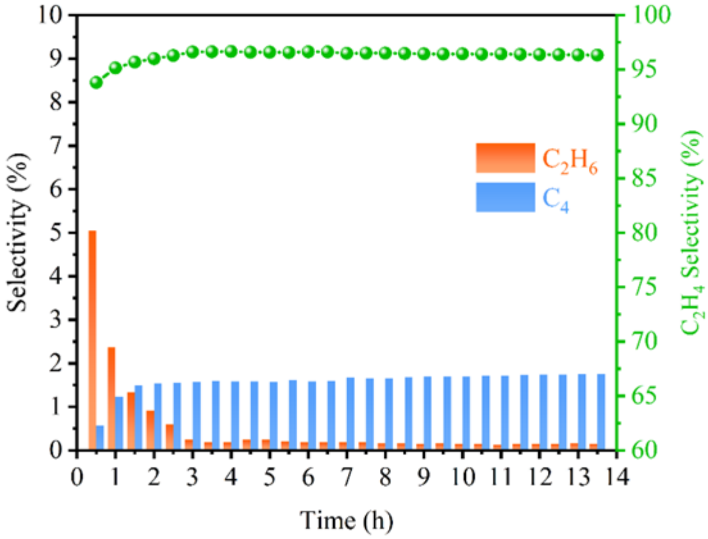

Figure S46. Selectivity vs. time of Cu-F in a 25 cm^2 flow-cell. Measured using a three-electrode flow cell (25 cm^2) in 1 M KOH at room temperature under 1 mol% C_2H_2/C_2H_4 flow (20 ml min^{-1}). A constant current density is set as 40 mA cm^{-2}. The results are presented without iR compensation.

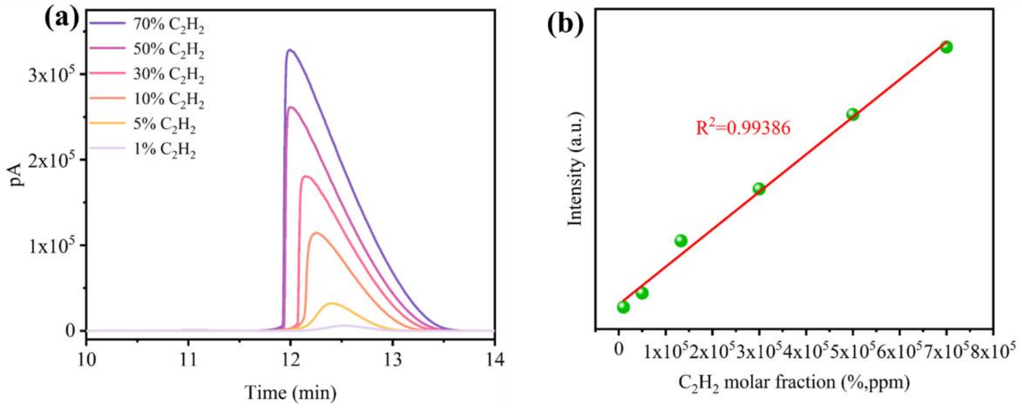

Figure S47. (a) Chromatogram and (b) the corresponding standard curve of C_2H_2 with different volume fractions for the quantitation of C_2H_2 conversion.

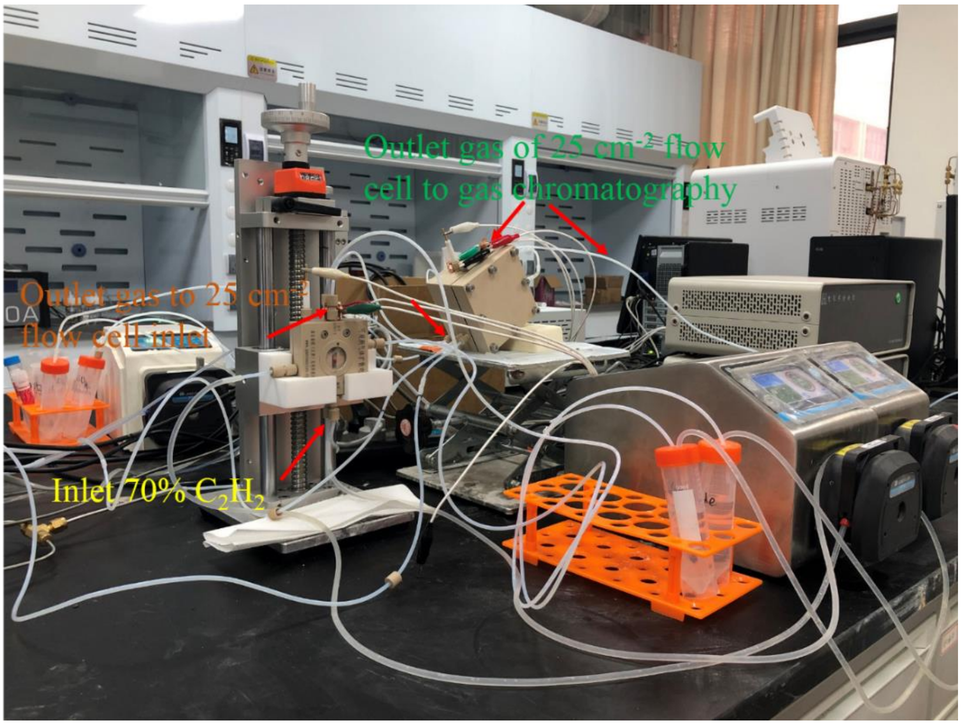

Figure S48. The tandem device composed of a 1 cm^2 and a 25 cm^2 flow-cell. The designing is based on the capacity of the 1 cm^2 flow cell to deal with high concentration C_2H_2 feed gas, and the ability of the 25 cm^2 flow cell for converting residual C_2H_2 at low concentration.

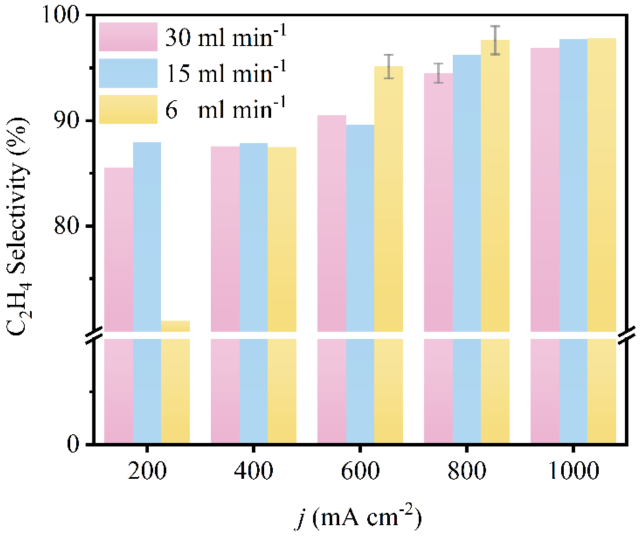

Figure S49. C_2H_4 selectivity vs. current density at different flow rate. Measured using a three-electrode flow cell (1 cm^2) in 1 M KOH at room temperature under 70 mol% C_2H_2/Ar flow. The results are presented without iR compensation. The maximum measurement error is ±4.7%.

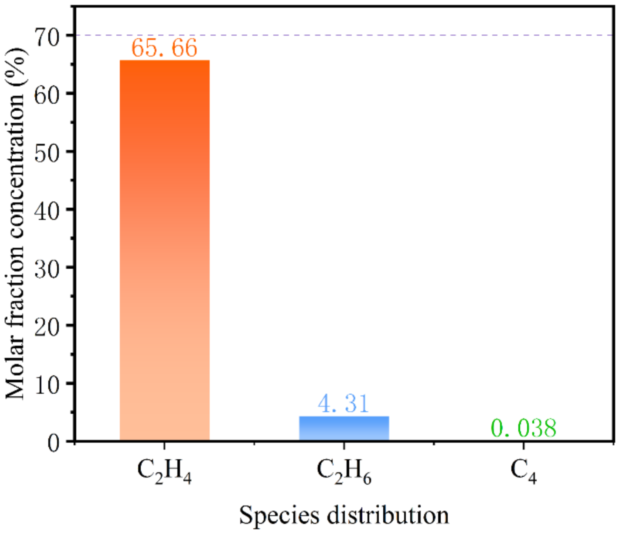

Figure S50. The carbon distribution in the outlet gas of the tandem device, which indicates a negligible carbon loss.

Table S1. Element contents in the halogen-containing copper catalyst measured by XPS.

Materials	Cu (atomic %)	X (F, Cl, Br, I) (atomic %)
Cu-F	84.4	15.6
Cu-Cl	87.0	13.0
Cu-Br	89.3	10.7
Cu-I	89.9	10.1

Table S2. Capacitance, surface roughness factors and electrochemical surface areas (ECSA) for Cu-X(F, Cl, Br, I) and Cu NP

Catalyst	C_{dl} (mF cm^{-2})	C_s[a] (μF cm^{-2})	ECSA (cm^2)
Cu-F	2.5		86.2
Cu-Cl	2.7		93.1
Cu-Br	2.8	29	96.6
Cu-I	2.9		100
Cu NP	3.3		113.8

[a] C_s the corresponding smooth polycrystalline Cu electrode.

Table S3. The comparison of acetylene semi-hydrogenation performance via electro-catalytic process

Catalysts	Gas (%)	$j_{C_2H_4}$ (mA cm^{-2})	FE $_{C_2H_4}$ (%)	C_2H_2 Con. (%)	C_2H_4 Sel. (%)	Stability (h)	Reference
Cu-F	C_2H_2:Ar= 70:30	~-764.6	92.39	~32.6 (30 ml min^{-1})	~94.47 (30 ml min^{-1})	43 (200 mA 30 ml min^{-1})	This work
	C_2H_2:C_2H_4:Ar =1:20:79	~-0.76	47.51	100 (20 ml min^{-1})	~96.29 (20 ml min^{-1})	13.5 (1.6 mA cm^{-2} 20 ml min^{-1})	This work
ED-Cu NPs	100% C_2H_2	~-298.5	99.5	~8 (30 ml min^{-1})	100 (30 ml min^{-1})	54 (200 mA 30 ml min^{-1})	Nat. Sustain. (2023) Doi:10.1038/s 41893-023-

							01084-x
Cu NDs	100% C_2H_2	~-350	~95.9	/	/	/	Nat. Commun. 14, 2137 (2023)
	C_2H_2:C_2H_4:Ar = 0.5:20:79.5	~-1.65	~88	99.9 (10 sccm)	92.6 (10 sccm)	70 (-1.9 mA 10 sccm)	
Cu dendrites	100% C_2H_2	~-134	96	/	/	12 (-50 mA cm^{-2} 20 sccm)	Nat. Catal. 4, 557-564 (2021)
	C_2H_2:C_2H_4=1:99	/	/	99.9 (20 sccm)	97 (20 sccm)	120 (-2 mA cm^{-2} 20 sccm)	
LD-Cu	C_2H_2:Ar= 5:95	~-65.0	74.9	/	/	/	Nat. Catal. 4, 565-574 (2021)
	C_2H_2:C_2H_4:Ar = 0.5:20:79.5	/	/	99.1 (1 ml min^{-1})	93.2 (1 ml min^{-1})	5	
NHC-Cu	100% C_2H_2	~-159	98	/	96 (20 ml min^{-1})	80 (-30 mA cm^{-2} 20 ml min^{-1})	Nat. Commun. 12, 6574 (2021)
	C_2H_2:C_2H_4=1:99	/	/	99.7 (10 ml min^{-1})	99 (10 ml min^{-1})	100 (4 mA cm^{-2} 10 ml min^{-1})	
Cu MPs	Saturated by C2H2	~-13.5	~50	9.3 (2.4 ml min^{-1})	80 (2.4 ml min^{-1})	100 (12 mA cm^{-2})	Nat. Commun. 12, 7072 (2021)
SA-Ni-NC	100% C_2H_2	~-84.2	~91.3	/	/	12 (30 mA cm^{-2} 20 ml min^{-1})	J. Mater. Chem. A 10,6122(2022)
	C_2H_2:C_2H_4=1:99	/	/	97.4 (10 sccm)	~99 (10 sccm)	8 (1.6 mA cm^{-2} 10 sccm)	
Cu SA/NC	20% C_2H_2	-100 mA cm^{-2}	87.5	/ (10 ml min^{-1})	/ (10 ml min^{-1})	15 (-100 mA cm^{-2} 10 ml min^{-1})	Angew. Chem. Int..Ed. e202307848 (2023)
	1% C_2H_2	-100 mA cm^{-2}	/	99 (-100 mA cm^{-2} 5 sccm)	97 (-100 mA cm^{-2} 5 sccm)	8 (-100 mA cm^{-2} 5 sccm)	

Table S4. The Ru resistances measured at working conditions in electrode system.

Electrode system	Catalysts	Resistances (Ω)	Electrode area (cm^2)
Three electrode system	Cu-F	1.1	1
	Cu-Cl	1.2	
	Cu-Br	1.2	
	Cu-I	1.2	
	Cu NP	1.2	
Two electrode system	Cu-F	9.4	
Three electrode system	Cu-F	1.5	25
Two electrode system	Cu-F	9.7	
Three electrode system	Cu-F	1.1 @50 mol % C_2H_2<br>1.1 @ 5 mol % C_2H_2	1
